# Baseline predictors of Internet‐based neuropsychological testing for amnestic mild cognitive impairment

**DOI:** 10.1002/alz70861_108765

**Published:** 2025-12-23

**Authors:** Sarah Wuesthoff, Kirsten MacDonnell, Basiru Dawda, Meghan K Mattos

**Affiliations:** ^1^ University of Virginia, Charlottesville, VA USA; ^2^ University of Virginia.edu, Charlottesville, VA USA

## Abstract

**Background:**

Identifying and diagnosing adults with mild cognitive impairment (MCI), especially those at known risk for Alzheimer’s disease, such as amnestic mild cognitive impairment (aMCI), is critical to preventing further cognitive decline. Internet‐based recruitment offers a promising, scalable strategy to reach older adults with cognitive concerns, yet predictors of MCI status derived from remote neuropsychological testing remain underexplored. This study examines potential predictors of Internet‐based neuropsychological test scores indicating aMCI for older adults with subjective cognitive concerns and enrolled in an Internet‐based randomized controlled trial (RCT).

**Method:**

*SHUTi MIND* is an ongoing, two‐year RCT evaluating an online insomnia intervention for older adults with subjective cognitive concerns. Participants were recruited online and screened using the Telephone Interview for Cognitive Status. At baseline, participants completed remote CANTAB neuropsychological testing and self‐reported questionnaires, which included questions about sociodemographic, modifiable (e.g., smoking, obesity, hypertension, diabetes, depression), and social/environmental determinants (e.g., education, head injury, social contact) included in this analysis. Predictors were binary‐coded (yes[1]/no[0]) using literature‐based cutoffs and aggregated into two indices: modifiable risk factors (0‐5) and social/environmental determinants (SEDs, 0‐3). aMCI status was defined using remote neuropsychological criteria. Group differences (aMCI+ vs. aMCI‐) were assessed with t‐tests and chi‐square analyses. Binary logistic regression modeled aMCI status using the risk indices.

**Result:**

Of 156 participants, 51.9% (*n* =81) met criteria for aMCI. Diabetes was the only individual predictor significantly associated with group membership (*p* =0.048), with fewer aMCI+ participants endorsing diabetes (*p* =0.048). Participants with 1‐4 modifiable risk factors had significantly lower odds of aMCI (ORs 0.07–0.18, *p*s<0.05), suggesting a non‐linear relationship. SED factors were not significant predictors (*p* =0.487). The overall logistic regression model was statistically significant (*p* =0.008) with good fit (Hosmer‐Lemeshow *p* =0.672).

**Conclusion:**

In this Internet‐recruited sample, aggregated modifiable risk profiles‐ but not SEDs‐ were associated with lower odds of aMCI as identified by online neuropsychological testing. These findings highlight the potential utility and challenges of digital recruitment and assessment approaches for identifying early cognitive impairment in older adults. Replication in larger, clinically diagnosed cohorts is needed to expand upon these findings and explore novel approaches to recruitment in this hard‐to‐reach population.

